# Cytokine Overproduction and Immune System Dysregulation in alloHSCT and COVID-19 Patients

**DOI:** 10.3389/fimmu.2021.658896

**Published:** 2021-06-02

**Authors:** Andrzej Lange, Janusz Lange, Emilia Jaskuła

**Affiliations:** ^1^ Hirszfeld Institute of Immunology and Experimental Therapy, Polish Academy of Sciences, Wroclaw, Poland; ^2^ Lower Silesian Center for Cellular Transplantation with National Bone Marrow Donor Registry, Wroclaw, Poland

**Keywords:** alloHSCT immunology, COVID-19 immunology, cytokine overproduction, immunogenetic profiling, monocytic-marrow derived suppressor cells, TCR beta repertoire

## Abstract

The COVID-19 pathomechanism depends on (i) the pathogenicity of the virus, (ii) ability of the immune system to respond to the cytopathic effect of the virus infection, (iii) co-morbidities. Inflammatory cytokine production constitutes a hallmark of COVID-19 that is facilitated by inability of adaptive immunity to control virus invasion. The effect of cytokine release syndrome is deleterious, but the severity of it depends on other confounding factors: age and comorbidities. In this study, we analyze the literature data on the post-transplant course of allogeneic hematopoietic stem cell transplanted (alloHSCT) patients, which is affected by generated inflammatory cytokines. The sequence of events boosting cytokine production was analyzed in relation to clinical and laboratory data highlighting the impact of cytokine generation on the post-transplant course. The collected data were compared to those from studies on COVID-19 patients. The similarities are: (i) the damage/pathogen-associated molecular pattern (DAMP/PAMP) stage is similar except for the initiation hit being sterile in alloHSCT (toxic damage of conditioning regimen) and viral in COVID-19; (ii) genetic host-derived factors play a role; (iii) adaptive immunity fails, DAMP signal(s) increases, over-production of cytokines occurs; (iv) monocytes lacking HLADR expression emerge, being suppressor cells hampering adaptive immunity; (v) immune system homeostasis is broken, the patient’s status deteriorates to bed dependency, leading to hypo-oxygenation and malnutrition, which in turn stimulates the intracellular alert pathways with vigorous transcription of cytokine genes. All starts with the interaction between DAMPs with appropriate receptors, which leads to the production of pro-inflammatory cytokines, the inflammatory process spreads, tissue is damaged, DAMPs are released and a vicious cycle occurs. Attempts to modify intracellular signaling pathways in patients with post-alloHSCT graft vs host disease have already been undertaken. The similarities documented in this study show that this approach may also be used in COVID-19 patients for tuning signal transduction processes to interrupt the cycle that powers the cytokine overproduction.

## Introduction

SARS-CoV-2 is a new virus, we do not know much about it, but like other RNA genome viruses, it jumps between species, and must adapt to each new environment, e.g. learning how to evade the host immune system. Interaction between immunity and the virus plays a special role. The affected organs in COVID-19 patients may come under friendly fire from unleashed inflammatory cells (1), bringing the risk of fatality. If adaptive immunity fails, the virus expands, pyroptosis is frequent, proinflammatory cytokines are released from damaged epithelial alveolar cells and macrophages, and in consequence the inflammatory process spreads (2). The released cytokines attract monocytes, which in inflammatory environment have increased phagocytic activity. The level of monocyte activation is reflected by a high serum ferritin level in severe COVID-19 cases (3), and the organ damage spreads in a vicious cycle of events. There are several examples in human pathology which document the costs paid to eliminate an infective organism. To eliminate the virus, the host’s own cells are frequently killed, and in addition, the inflammatory process damages tissues in an innocent bystander mode. In severe COVID-19, within the pathological process, several organs are damaged, not necessarily those directly invaded by the virus. The essence of this pathology is a massive release of cytokines – the cytokine storm – which is associated with life-threatening complications.

Cytokine release syndrome is seen in several pathologic situations, but that in patients having acute graft vs host disease after allogeneic hematopoietic cell transplantation (alloHSCT) was so impressive that Ferrara and his colleagues coined the name cytokine storm (4). Therefore, to learn more about the manifestation and consequences of cytokine release syndrome, we looked at this phenomenon from the perspective of an observer of alloHSCT patients. In this study, clinical consequences and a laboratory description of the events after alloHSCT are discussed in relation to the course of COVID-19 in which cytokine release syndrome is a risk factor of poor prognosis.

In alloHSCT patients the primary proinflammatory event triggers a chain of inflammatory sequelae. This is shown in this paper in the context of corresponding information on COVID-19 patients. The latter attempt was undertaken due to our belief that the experience obtained looking after alloHSCT patients may help, by analogy, in designing the treatment approach in COVID-19 patients (**Table 1**). Unfortunately neither Tocilizumab (46) nor Ruxolitinib (47, 48) was found to be effective. Mesenchymal stem cells (MSC) use is still promising but lacks approval. Several U.S. Food and Drug Administration approved clinical trials on the use of MSC in COVID-19 patients are ongoing (49).

**Table 1:** The clinical findings and laboratory data registered in patients at risk of cytokine storm after alloHSCT and in those with severe COVID-19.

	alloHSCT	COVID-19 symptomatic cases
Proinflammatory environment	Toxicity, engraftment syndrome, GvHD, recurrent infections (5, 6),	Cytopathic effect on SARS-CoV-2 infected cells (7)
CD14+HLADR-	Increased proportion in early post-transplant period affecting the long term survival, and GvHD (**Figure 1**) (8–10)	Increased proportion in severe COVID-19 (11, 12)
IL-6	High level in post-transplant period (13, 14)	High level in severe COVID-19 (15–17)
lymphocytopenia	Frequent at the time of hematologic recovery (18)	Present in 80% of cases (11, 12, 19)
	-associated with cytokine fluctuations (5)	
	-risk factor of aGvHD (20) and a*GvHD* following DLI (21)	
Immunogenetic profiling	IFN gamma +874 A (13 CA repeats) allele	
	Is associated with EBV and CMV (22, 23) reactivation and GvHD (24, 25)	Is associated with SARS caused by coronavirus (26)
	IL-6 -174 G allele	
	risk factors for GvHD (14)	Postulated to be associated with COVID-19 susceptibility (27)
CMV reactivation	Frequent (28–30)	Single case reports and the negative impact of chronic CMV infection is suggested (31)
EBV reactivation	Frequent (28)	Frequent (32)
Immune dysregulation syndromes	HLH in 4.3% of cases, with 85.5% mortality (33, 34)	HLH and TMA are frequent in COVID-19 patients (16, 35–38)
	TMA in 10 to 20% of cases (39) with 44% mortality (40)	
Main target organ(s) when cytokine storm develops	Multiorgan involvement as a result of plasma cascade dysregulation, toxic internal organ damage and alloreactivity (41)	Acute respiratory distress syndrome (42)
		Plasma cascade dysregulations (43)
Extended symptomatology	Mostly due to alloreactivity and prolonged immunosuppression	“long tail” COVID-19 with multiorgan symptomatology and different mechanisms leading to patients disability (44, 45)

alloHSCT, allogeneic stem cells transplantation; GvHD, Graft versus Host Disease; DLI, Donor Lymphocyte Infusion; HLH, hemophagocytic lymphohistiocytosis; TMA, thrombotic microangiopathy.

Analyzing the cytokine overproduction in COVID-19 we are aware of other factors which independently or in concert with cytokine overproduction make infected people more vulnerable. The recent data on the case fatality rate in COVID-19 patients in China shows that 10.5% of people having cardiovascular disease, 6% suffering from hypertension and 7.6% form diabetes who were diagnosed with COVID-19 died (50).

The common denominator of these diseases is the presence of vascular pathology. Local and systemic inflammation which characterizes COVID-19 activate and damage endothelium. It is shown by elevated level of von Willebrand Factor (VWF) in blood The inflammatory process damaging endothelium facilitates microangiopathy (51). The mechanism of the latter pathology resembles that seen in sepsis.

Microangiopathy may drive COVID‐19 progression in which comorbidity adds to the risk of COVID-19 outcome.

Thus in the patients with comorbidities inflammatory cytokines addressing already damaged tissues may operate at a lower concentration level. Indeed the level of IL-6 in COVID-19 patients was reported to be lower than in other diseases attributed to cytokine overproduction (52–56). The serum level itself, however, cannot be used solely for validation the role of IL-6 in the pathomechanism of COVID-19 as the deteriorating effect of this inflammatory cytokine depends also on the susceptibility of the targeted organ which is higher if concomitant disease exists (57). It has been already documented in a number of studies that the high level of IL-6 is a predictive factor of poor outcome of COVID-19 (53, 56). In a recent study 1,484 patients with suspected or confirmed COVID-19 were investigated with a conclusion that the levels of IL-6 and TNF alpha in serum at presentation are predictive of COVID-19 survival and mortality, independently of demographics and comorbidities (52).

Comorbidities especially those associated with the endothelial cells damage depicted by elevation in serum of VWF factor include cardiovascular diseases, hypertension and diabetes (58) makes the vasculature more susceptible to thrombotic events thus shaping the course of the disease. The primary event is the same as in the patients lacking comorbidities i.e. overproduction of inflammatory cytokines (59).

The similarities discussed above are due to the fact that the cytokine storm outcome in severe COVID-19 and in alloHSCT patients is very similar as in both situations disruption of homeostasis of the immune system determines the pathology. However, at the initiating stage the pro-inflammatory stimulation is viral and sterile in COVID-19 and alloHSCT, respectively. For SARS-CoV-2 viral lung damage is decisive for the fate of patients but not in alloHSCT patients. It has been recently discussed whether in COVID-19 patients the impact of SARS-CoV-2 on mitochondrial function plays a role by boosting the inflammatory process. It is also postulated that mitochondrial damage may contribute to the symptoms of “long COVID-19”. In the course of COVID-19 mitochondria are exposed to damage, which may result in a low energy potential of the affected cells and from patients’ perspective in long term observed fatigue and a lack of energy (44). This is also unique for COVID-19 patients (45).

Damage-associated molecular patterns (DAMPs) and pathogen-associated molecular patterns (PAMPs) stimulate membrane or cytosolic pattern recognition receptors (PRRs). These receptors are present in a number of cells, among which the most potent cytokine producers are macrophages and T cells (60). PRRs sense products released from dying cells and some structures of bacteria, fungi, as well as viral nucleic acids including RNA (61). In response, a number of pro-inflammatory cytokines are released, including G-CSF, IL-1,TNFalpha, IL-33, IL-23, IL-17 (5, 62). In alloHSCT patients receiving conditioning regimen (chemo-radiotherapy), pro-inflammatory cytokines are generated in response to direct cells damage (DAMP) as well as to microbiome (PAMP) being released to circulation from the gut losing its integrity. Several organs are damaged, apoptosis is frequent, and the inflammatory process exacerbates it (62). The consequences of conditioning regimen toxicity in alloHSCT patients are similar to those resulting from the cytopathic effect of SARS-CoV-2 and direct recognition of viral RNA by cytosolic RIG-1 like receptors (RLR) (63–65).

The peculiar feature of SARS-CoV2 is its ability to delay the IFN beta response (66), which facilitates the virus invasion and damage of targeted epithelial and endothelial cells. In consequence, DAMPs are released, which in concert with virus particles stimulate production of pro-inflammatory cytokines. In addition, monocyte-macrophages engulf viruses, release cytokines in response but also may serve as a virus reservoir (67) for further virus surge, which keeps the cytokine release ongoing. Among them TNF alpha plays a significant role due to the activation of NF-kappaB signaling pathways (pro-inflammatory) and facilitation of apoptosis and other forms of cell death (68). TNF alpha together with IFN gamma abolishes germinal center formation in the lymph nodes (68). In this situation the B cell response is restricted to germline-encoded low affinity antibodies and lacks cooperation with germinal center T follicular helper cells, which secure antibody production of high affinity, offering long term protection (69) (**Figure 3**).

IFN gamma and TNF alpha drive macrophages of an abundant inflammatory phenotype to the lung in severe COVID-19-cases (75). As a result the tissue affected by SARS CoV2 is susceptible to inflammatory cell death, which includes apoptosis (SARS-CoV-2 encoded accessory protein ORF3a can induce apoptosis) (76) as well as pyroptosis and necroptosis. There are forms of inflammatory cell death which reflect the inability of the cells to eliminate the pathogen. It is a suicidal action which triggers the inflammatory response and activates the immune system (77). Mechanistically, the JAK/STAT1/IRF1 axis is involved, leading to caspase-8/FADD-mediated PANoptosis (78) (**Figure 3**).

Inhibition of both IFN gamma and TNF alpha was effective in reduction of inflammatory cell death in experimental models of sepsis, hemophagocytic lymphohistiocytosis (HLH), and cytokine shock. This shows that TNF alpha and IFN gamma exert their vicious effect in several situations associated with immune system dysregulation (78) which may also happen in alloHSCT patients.

**The engraftment process** is shortly preceded by the generation of stress cytokines, especially IL-6, which activate transplanted progenitor cells to settle in the bone niches to start regeneration of hematopoiesis. If there is massive production of cytokines, engraftment syndrome develops, being clinically severe (79), with fever, skin rash, and weight gain (80).

Acute graft-versus-host disease (aGvHD), which results from alloreactivity of transplanted lymphocytes directed against host body tissues, constitutes the third consecutive phase in the post-transplant course in which cytokines are generated in a great amount. The level of cytokine release at aGvHD is as high as it was during the preceding phases when cytokines were generated in response to conditioning regimen toxicity.

Early post-transplant toxicity increases the risk of aGvHD and if the alloreactivity is clinically manifested the production of pro-inflammatory cytokines, being already high at the engraftment syndrome phase, is accelerated. A cytokine storm is fully manifested. Chronic production of pro-inflammatory cytokines dysregulates the cytokine network, which instead of returning to homeostatic balance goes into immunopathology. Chronic production of pro-inflammatory cytokines advances along the natural history of alloHSCT patients (4), reaching in 10% to 20% of cases the stage in which homeostasis is totally broken, resulting in manifestation of life-threatening complications [i.e. hemophagocytic lymphohistiocytosis (HLH) and thrombotic microangiopathy (TMA)] (39). In COVID-19 patients pro-inflammatory cytokine production spikes if adaptive immunity fails in virus eradication (2). These patients also come to the stage when homeostasis is broken, being at high risk of TMA and HLH (81).

## Lymphocytes and Monocytes in the Pro-Inflammatory Environment

Lymphocytes are low in numbers in COVID-19 and in the course after alloHSCT. Low counts of lymphocytes (11) and functionally impaired monocytes (8) make the patients susceptible to infection/reactivation of infective microorganisms. Effective T cell activation in response to a given pathogen needs the presence of a large number of lymphocytes to have a chance in finding cell having CDR3 able to align with the presented epitope (82). If it happens T cells may activate B cells having compatible membrane bound immunoglobulins named B cell receptor. and CD8+ cells which expanding constitute a population of cytotoxic cells. Low numbers of CD8+ and B cells seen in COVID-19 cases are indicative of outcome (83) and in alloHSCT patients lymphocytes are usually low and B cells reconstitute rather late after alloHSCT (18, 84).

Prolonged post-transplant lymphocyte recovery is associated with poor survival (85). The pace of the recovery depends on the number of CD34+ cells infused (86) and the early recovery of T cells associates with overall and event free survival (87). Therefore, the immune system of recipients after transplantation depends on the immune competence of the donors as well as richness in stem cells of the inoculum used. A high number of CD34+ cells in the transplant material is associated with a high number of naïve cells 4 weeks after transplant, especially in patients not experiencing EBV or HHV6 reactivation (88). The repertoire of TCR alpha/beta lymphocytes in the post-alloHSCT patients is restricted if they received a transplant from adults with T cell depletion, but it may appear quite well if cord blood is transplanted (89, 90). However, the latter association may be simply due to younger age of the recipients who in cord blood transplantation they are usually small. The diversity of lymphocytes in young individuals is much greater than in older ones due to the constant supply of recent thymic emigrants. Naïve cells pool increases what makes recognition of strange antigens possible. These observations help in understanding different post-transplant medical history in adults and children. In COVID-19 children cases lymphopenia is rather rare specific B memory cells and neutralizing antibodies in the blood are present (91).

From 6 months to 3 years after alloHSCT TCR repertoire diversity becomes close to that seen in normals if patients were transplanted from adults (89, 90). Including a high representation of the immunodominant clones which cover a great part of the homeostatic space (92). The immune response concentrate frequently in older individuals on a restricted number of epitopes, likely neglecting others. In the HSCT recipients in contrast with normal individuals, the number of clones which had already been annotated to viral infections is greater and they cover more space. Within identified immunodominant clones in 27 situations, CDR3 sequences found had already been annotated to public or viral epitopes (93) among which CMV plays a main role TCR annotated to CMV consume much of the homeostatic space covering cells with TCR specificity so far not exploited (94).

The above data show that the patients with dysregulated immunity have their epitope recognition potential focused on the response toward repeatable infections and may utilize TCR gamma delta cells in immunosuppressive conditions.

Monocytes appear in the blood early after alloHSCT. They are in the front line of microbial defense, alerting the immune system by releasing cytokines. The cells engulf infectious particles, break them down, and bring them to the cell membrane, which makes the presentation to T lymphocytes possible (95), initiating the adaptive immune response. If it fails or is not good enough, the local inflammatory process accelerates, attracting more monocytes, which migrate to the site of inflammation from the periphery according to the CCL2-CCR2 axis (96). If T cells respond well in the priming process, the specific clone(s) expand, the affected organ is cleared, and the fight is successful. There are, however, several obstacles on the way: (i) monocytes if low in number or lack CCR2 are not recruited well, which increases the affected organ pathology as is seen in the mouse model of influenza in which a lack of CCR2 on monocytes increases the organ pathology (97); (ii) monocytes are present but lymphocytes fail, not recognizing the epitopes presented by antigen presenting cells due to the low number or poor repertoire of T cells, and (iii) the appropriate cells are present but the immune response is paralyzed by monocytic-myeloid derived suppressor cells. These possibilities may affect the outcome of viral infection, as seen in COVID-19 patients but also valid in alloHSCT patients. Viral reactivation(s) is common and if it happens facilitates the aGvHD process (98). In aGvHD, monocytes that colonize the skin present skin or foreign antigens, which might also be of virus origin, to allogeneic T cells. In intestine GvHD, both alloreactivity and *Cytomegalovirus* (CMV) infection accelerate each other (99). Monocytes sense through Toll-like receptors a wide array of damage-associated patterns from dying cell debris through viral RNA to bacterial LPS (100). Once being activated by sensing the primary sterile signal, they may be further boosted by concomitant infections or vice versa. The described pathomechanism is understandable, but it is very difficult to manage as the only way to break the spiral of pro-inflammatory monocyte activity is the effective action of the adaptive immunity response, which is poor in inflammatory conditions and the situation usually affects people who are immunosuppressed because of the actual clinical situation (transplantation) or due to the characteristic of a primary disease (viral, autoimmune) (101).

### Monocytic-Myeloid Derived Suppressor Cells (M-MDSC)

As a response to pro-inflammatory situation, CD14+ cells lacking HLADR on the membrane appear in the blood. Downregulation of HLADR on the membrane of monocytes is driven by IL-6 and G-CSF, which act in concert with other pro-inflammatory cytokines (9, 102, 103). A lack of HLADR antigen on the monocyte membrane makes the cells suppressive with the aim to control the damaging inflammatory process (9). CD14+HLADR- cells represent monocytic myeloid-derived suppressor cells (M-MDSCs). These cells suppress the response to infection but being non-specific may hamper immune surveillance of cancer (104) and exert a negative impact on the immune response. M-MDSCs are weak in phagocytosis but efficient in immunosuppression, exerted by generation of reactive oxygen species, nitric oxide (105). The frequency of CD14+HLADR- (M-MDSCs) is increased in the peripheral blood after alloHSCT, especially in patients with GvHD (9) and bacterial infections (106, 107). CD14+HLADR- cells were primarily found in septic cases and their suppressor activity may lead to immune paralysis (107). Sepsis, one of the major complications of the patients after alloHSCT, seen also in those with COVID-19, is clinically apparent when infection is not under control and a destructive immune response leads to overwhelming pro-inflammatory activity (1). Indeed, in COVID-19 patients the number of CD14+ cells lacking HLADR is increased and remains so through the course of the disease (12, 108). It was also reported that an increase in the proportion of CD14+ which lack HLADR positivity is associated with the progression in the course of COVID-19 (12).

Therefore, attempting a comparison between alloHSCT patients and those with COVID-19, evaluation of CD14+HLADR- cells in both these situations was of great importance. We recently analyzed the impact of CD14+HLADR- cells on survival of patients transplanted in our institution. The higher values of these cells determined on the post-transplant day 30 have a negative impact on survival and the patients they died succumb more frequently of infections than of other causes including GvHD and relapse, survived shorter and had still higher values of CD14+HLADR- cells than those which also died but were from the low CD14+HLADR- group (8). Therefore, CD14+HLADR- cells measurements on the post-transplant day 30 depict the cases they experienced overproduction of inflammatory cytokines what encumbers further history of the disease.

**Figure 1:**
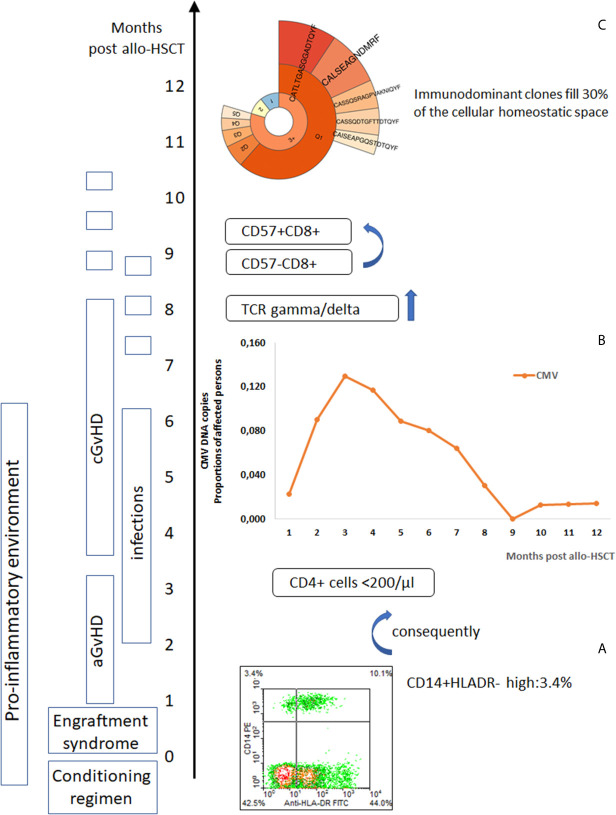
Post-alloHSCT chain of events affecting immune system competence. **(A)** Post-alloHSCT toxicity induces a pro-inflammatory environment with an increase in blood monocytic-marrow derived suppressor cells (CD14+HLADR-) (8), **(B)** Herpes viruses reactivate (CMV reactivation events in post-transplant period) (28, 29), in the peripheral blood lymphocytes CD8+CD57+ cells increase (110) – T cell repertoire skewed to highly differentiated T cells effective against chronic infection epitopes but neglecting new challenges, **(C)** TCR gamma/delta cells reprogrammed by CMV reactivation appear frequently in the blood (prevalence of deltaV2 negative cells) (111), immunodominant clones expand (93).

**Figure 2:**
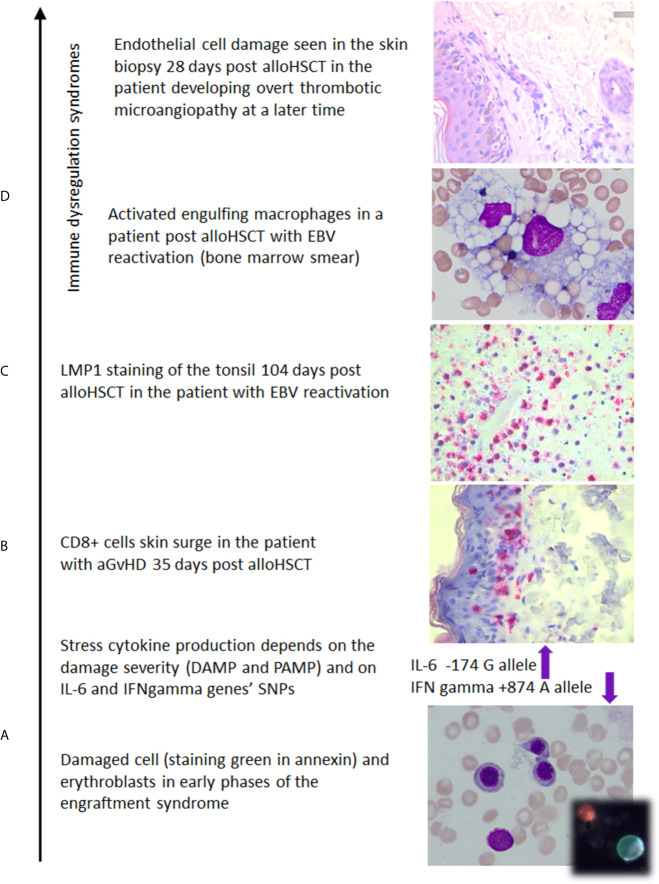
The photos illustrate (own documentation): **(A)** damage of the marrow which induces IL-6 and release of other stress cytokines whose level is modified by SNP polymorphic features (14, 24, 25, 112, 113), **(B)** skin biopsy with CD8+ cell epithelium colonization in aGvHD case (114), **(C)** EBV reactivation documented on 104^th^ day after transplant (23), **(D)** macrophages in a patient with hemophagocytic syndrome, the skin biopsy documenting early endothelial cell damage in the patient developing overt thrombotic microangiopathy at a later time.

Our and others studies (8, 109) provide a compelling evidence that an increase of CD14+HLADR- suppressor cells-in blood put patients in danger of life threatening infections. In that situation, microbial invasion is not prevented by the specific immune response instead of an overwhelming inflammation takes place, resulting in dysregulation of immunity (**Figures 1**, **2**). This is a crucial element aggravating the risk of death, especially when the primary response is triggered by tissue injury due to toxicity, as it is in alloHSCT patients, or by the cytopathic effect of a virus exerted long before the immune system is ready to respond, as is seen in an unknown pathogen infection such as COVID-19 for example. In a situation when toxicity but not microbial invasion triggers the immune response with inflammation at first as seen early after alloHSCT, CD14+HLADR- cells appear and their suppressor activity may facilitate microbes including viruses to sneak through the immune system barrier. If M-MDSCs (CD14+HLADR-) are too active, they may suppress the immune response to the microbes, affecting patients with aGvHD (107). For that reason Herpesvirus reactivation is frequent in patients after alloHSCT and also in those suffering from COVID-19 due to the presence of a pro-inflammatory environment (31) with the consequences discussed above. CD14+HLADR- cells associated immunosuppression developed to counter-balance pro-inflammatory status facilitates microbial complications to appear. The CMV reactivation period starts about the time of the engraftment syndrome characterized by stress-cytokine production. The proportion of patients with CMV reactivation remains high up to 6 months after transplant and then declines (**Figure 1**). The *Epstein-Barr virus* (EBV) prevalence curve is similar to that of CMV early after alloHSCT but EBV is even more frequently seen (likely due to CMV infection prophylactic measures) by the end of the first year of post-transplant observation. More than 60% of patients experienced reactivation of at least one of herpesvirus reactivation in the 6-month post-transplant period (28, 29, 115).

### The Impact of Chronic CMV Infection on Blood Lymphocyte Subpopulations

Chronic CMV infections blows up the immune system, directing the response toward the virus at the expense of the response to other microbial challenges (116). This is also seen at the level of the peripheral blood lymphocyte profile.

Herpes virus reactivation depends on the immune system competence of the transplant donor. Reactivation of CMV repeatably stimulates the immune system to engage in CMV immunity while neglecting responsiveness against other antigens (29). This also has an impact on the balance between different lymphocyte subsets. The T lymphocyte profile shifts in chronic CMV infection from CD28+CD57- T cells toward highly antigen experienced CD8+ CD57+ cells (117). The latter cells occupy a substantial proportion of lymphocytes, leaving less homeostatic space for naïve cells and central memory cells, which are able to confront new antigenic stimulation (118). It is, however, individually dependent. HLA-A* 0101 individuals T cells response against CMV pp50 characterizes with a broader spectratype than that seen when CMVpp65 is targeted (91).

TCR gamma/delta cells may increase in proportion in several infections. Predominant increase of one out of two main subsets, having either a Vdelta 1 or Vdelta 2 chain, suggest the presence of a defined microbe behind the stimulation (119). Vdelta 2- cells are usually associated with CMV reactivation (111, 120). Whereas in CMV negative patients both subsets Vdelta 1 and Vdelta 2 accumulate at the similar level (121). Vdelta 1+ cells recognize stress-related antigens and also those characteristic for some pathogens including mycobacteria, influenza viruses, and EBV (122). Therefore, the results of detailed profiling of TCRgamma/delta indicate which pathogen may be involved (119, 122). In COVID-19 patients, gamma/delta T cells are low at the onset but increase in survivors during the later course of the disease. It is thought that they may be directly or indirectly (as antigen presenting cells) involved in the immunity against SARS-CoV-2 (123). The positive role of TCR gamma/delta cells is also seen in alloHSCT patients who enjoy better survival, having an elevated level of TCR gamma/delta cells and a lower incidence of bacterial and virus infections [reviewed by Handgretinger et al. (124)]. These data suggest that TCR gamma/delta cells are effective in patients with dysregulated immunity in which the adaptive immunity response is lowered due to the higher proportions of M-MDSCs in the blood. However, there are also some data pointing on the suppressive effect of M-MDSCs on TCR gamma/delta T cells (125).

**Figure 3:**
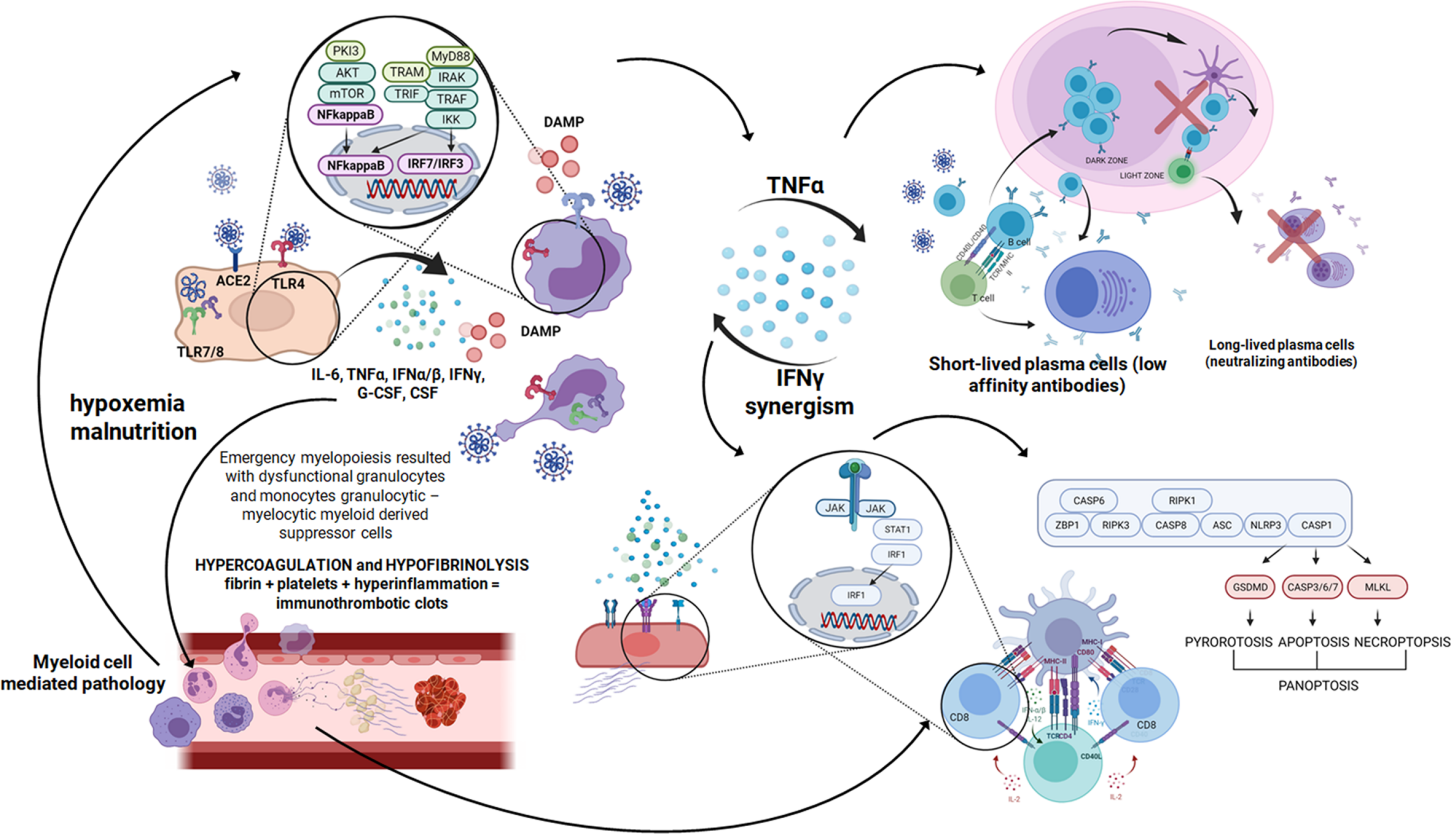
A cascade of events after SARS-CoV-2 infection starts with a few days time lag in which the virus replicates using a set of open frame genes in airway epithelial cells (70). Then pneumocytes are invaded and huge amount of DAMPS are released (71). After that innate immunity receptors recognize viral RNA and proinflammatory cytokines are produced using the NFkappaB signaling pathway. Pyroptosis is seen and proinflammatory cytokines build up an inflammatory response (72). Then macrophages, monocytes and polymorphonuclear leukocyte are recruited and activated in the inflammatory environment and the next wave of cytokines surges. In severe COVID-19 cases as a result of emergency myelopoiesis dysfunctional mature neutrophils and HLADRlow monocytes appear in the blood (73). In this complex situation the endothelial cell infection outcome contributes additionally to the inflammatory response. Endothelial damage leading to thrombolytic microangiopathy causes hypoxemia and malnutrition (43). The mTOR pathway is activated, which ends up with NFkappaB and massive cytokine release (74). Created in Biorender.com.

Granulocytes play a significant role in innate immunity response to pro-inflammatory cytokines (**Figure 3**). They produced reactive oxygen species contributing to tissue damage (126) as well as they affect plasma cascades increasing the risk of immunothrombotic clots formation (43). Similarly to the suppressive potential of monocytic-MDSC also granulocytic MDSC may play a role (127). They are released to the periphery in the course of emergency myelopoiesis driven by the proinflammatory environment and are dysfunctional, and may decrease T cell immune response (128).

Individual genetic variation in the immune system, especially gene expression variability, may affect the course of several diseases including COVID-19. The immune response is very much controlled by the genetic factors and one of the first impressive observation made in man was on the higher susceptibility to immunopathology in carriers of ancestral HLA A1-B8-DR3 haplotype (129). This is claimed to be due to the presence of linkage disequilibrium with some genes within the genome including the polymorphic feature of TNF alpha gene. This genome is associated with the presence of antinuclear antibodies under the environmental stress (130). This shows that the genetic features of inflammatory gens may influence the outcome DAMPs in COVID-19 and alloHSCT patients. While analyzing the risk of an overwhelming inflammatory response, the SNP polymorphism in the IL-6 gene is of interest. The *IL-6 G* allele situated at the promotor –174 position (rs1800795) is associated with higher IL-6 generation as opposed to the presence of the *IL-6 C* allele in the same position (131, 132).

Among alloHSCT receiving patients, those with high pre-transplant levels of IL-6 have higher frequency of transplant-related mortality as compared to their counterparts (13). This observation is supported by the genetic data showing that *IL-6 G* allele carriers have higher levels of IL-6 in the blood during the post-transplant period and if transplanted from a donor homozygous for the *IL-6 G* allele are at a high risk of severe aGvHD (14, 112, 113). When viral infections are considered, good IL-6 producers may benefit at the front line of infection (e.g. in the common cold) but not when the disease goes wrong, high IL-6 individuals are more susceptible to acute respiratory distress syndrome.

High levels of IL-6 or its read-out protein CRP in serum should alert the medical staff that the course of COVID-19 can take a turn for the worse (15). Immunogenetic profiling, performed in advance, may help in risk assessment of the course of the disease, which may be valid for alloHSCT as well as COVID-19 patients.

Interferon (IFN) gamma is the other master cytokine of the immune system, and its generation is modified by SNP profiles. Individuals homozygous for micro-satellite polymorphism of 12 CA repeats starting at position +875 (or having a T nucleotide at polymorphic position +874 - rs2430561) have a higher generation potential of IFN gamma in PBMC stimulated by mitogens than those having more than 12 CA repeats (or being +874 A) (133). Likely due to HSCT patients homozygous for 13 CA repeats being poor producers of this cytokine under stimulation, they are more susceptible to CMV and EBV reactivation (22, 23) and finally to GvHD. Notably, the low IFN gamma producers having the SNP *IFN gamma* +874 *A* allele were found – in a study carried out during the 2002–2003 SARS-CoV-2 epidemic outbreak – to be associated with susceptibility to SARS in a dose-dependent manner (26).

The data above suggest that the immunogenetic profiling of IL-6 and IFN gamma genes may be of use in weaving out the individuals at higher risk of cytokine storm while having COVID-19.

The grave experience of the global COVID-19 pandemic has focused attention on immunotherapy, which earned considerable publicity by proving effective in some cancer treatment approaches. The use of check-point inhibitors relies on the blockage of naturally occurring regulatory mechanisms releasing CD8+ cells from negative feedback control. Unfortunately, this approach, omitting pro-entropic rules, may result in auto-aggression. The constant uncontrolled activation of T cells results in over-production of cytokines with target organ injury and CMV reactivation. A similar scenario is seen in patients receiving CAR T cells in which cytokine storm plays a negative role with severe consequences (134). Cytokine storm is also a risk factor of fatal COVID-19. Therefore, in several clinical situations, cytokine storm as an effect of immune system dysregulation should be considered as a main threat and addressed in research to save lives. In gearing up cytotoxic mechanisms we should also know how to revert them. In this review, we have gone over the events occurring after alloHSCT. This approach was aimed at raising alloreactivity against cancer cells. Unfortunately, cytokine storm can make the procedure difficult for patients and, in a proportion, fatal. Tuning of the immune response is needed to have the target cell eliminated but not at the expense of organ injury. Immunosuppression and steroids used in alloHSCT cases should be rather individually adapted in relation to the actual competence of the immune system. In alloHSCT patients the level of CD14+HLADR- cells if increased may alert the medical staff that competence of the immune system reaches a dangerous state (8, 109)

The optimal option is to measure the immune response at all stages of treatment. To do so, we have to (i) implement immunogenetic profiling to stratify the patients at risk (IL-6, IFN gamma genotyping), (ii) follow the phosphorylation of the master kinases of the signaling pathways to understand the balance between signaling pathways important for keeping cells in homeostatic order (135), (iii) detect the methylation pattern of STAT3 and 5, which are associated with the potential of Treg cell and Th17+ cell generation. For that, next-generation sequencing (NGS) will be used, providing the tool for genetic work. Being under the pressure of pandemic threat, we all have to use our intellectual and laboratory potential to manipulate the immune system cautiously, based on the known facts. This will require international cooperation.

## Conclusion

Individuals confronted with prolonged stimulation of the immune system develop a mechanism to respond to the stressful situation and to keep the response under control (136). The innate immune response stays up-front to gain time needed for the adaptive immunity to develop. In several situations, adaptive immunity is not efficient enough due to host-derived or environmental factors. Inflammation overwhelms and the cytokine storm is full blown, presenting with fever, body fluid retention, malnutrition, and endothelial cell damage, facilitating disseminated intravascular coagulation. These symptoms manifest in the impairment of body homeostasis with poor blood oxygenation and malnutrition. Both are sensed, causing a switch in the cellular signaling pathways favoring the AKT/mTOR signaling at the expense of others (137, 138). The mTOR pathway, activated by pattern receptor ligation and sensing *via* HIF-1 poor tissue oxygenation, takes over other pathways, tilting the balance inside cells from steady state toward alert mechanisms (139). There is a parallel between HSCT recipients and COVID-19 patients in the pathological events mediated by the cytokine storm, suggesting that therapeutic approaches, developed in the context of HSCT, may prove beneficial in COVID-19.

The first cause in the COVID-19 pathomechanism is the viral cytopathic hit inducing the response of the immune system to the pathogen and damage-associated molecular patterns. The cytokine response to the stimuli determine the following steps. Toll-like receptor signaling by PAMP results in production of IFN I (IFN type I). This cytokine facilitates the priming of adaptive immunity cells (140). It is known that severe COVID-19 patients are poor in mounting an IFN I (INF type I and II and III response as assessed at the transcriptional level (141–143). This characteristic hampers the adaptive immunity potential, whose activity is facilitated by IFN(s). If the adaptive immunity is slow, PAMP and DAMP(s) release occurs (due to the cytopathic effect of the virus), activating the inflammatory response, which may be ameliorated by the adaptive immunity response eliminating the pathogen. Among the stress cytokines responding to DAMP(s) IL-6 plays a prominent role in the pathogenesis of overwhelming cytokine production in both severe COVID-19 and advanced aGvHD cases – the cytokine storm which causes the dysregulation of the immune system and deepens immunosuppression (52, 141, 144, 145). Keeping in mind the different mechanism leading to the overwhelming cytokine production, the outcome of that is similar in severely manifested alloHSCT (especially in those with high grade aGvHD) and severe COVID-19 (poor relay between innate and adaptive immunity). The level of serum IL-6 is high in both severe COVID-19 cases and in alloHSCT patients having high grade aGvHD, being predictive of an ominous outcome (14, 52–57, 141). The common clinical outcome results from dysregulation of immunity.

The pathomechanism of complications after alloHSCT and those of severe COVID-19 depend on the competence of the immune system which deteriorates with age (146) and underlying diseases (144). In both situations everything starts with the generation of stress cytokines, which may lead to the cytokine overproduction. This is the subject of the present study. Purposely we illustrated the laboratory findings with clinical pictures, which may help the health care providers in timely recognition of cytokine overproduction, which if present must be controlled effectively.

Bearing in mind the dissimilarities between these two clinical situations the message we wish to convey to readers is that the pathologic abnormalities when a cytokine storm erupts in response to the cell damage, viral or sterile, overwhelms the immune system with DAMPs and PAMPs, with the cytokine milieu being similar in both situations.

## Author Contributions

AL: study design, writing of the article, identification the papers for inclusion. JL: revising of the manuscript. EJ: gathering of data, co-writing the article, editing, and revising of the manuscript. All authors contributed to the article and approved the submitted version.

## Funding

Preparation of this publication was supported by L. Hirszfeld Institute of Immunology and Experimental Therapy, Polish Academy of Sciences.

## Conflict of Interest

The authors declare that the research was conducted in the absence of any commercial or financial relationships that could be construed as a potential conflict of interest.
